# Exhaustive Sampling of Docking Poses Reveals Binding Hypotheses for Propafenone Type Inhibitors of P-Glycoprotein

**DOI:** 10.1371/journal.pcbi.1002036

**Published:** 2011-05-12

**Authors:** Freya Klepsch, Peter Chiba, Gerhard F. Ecker

**Affiliations:** 1 Department of Medicinal Chemistry, University of Vienna, Vienna, Austria; 2 Institute of Medical Chemistry, Medical University of Vienna, Vienna, Austria; University of Houston, United States of America

## Abstract

Overexpression of the xenotoxin transporter P-glycoprotein (P-gp) represents one major reason for the development of multidrug resistance (MDR), leading to the failure of antibiotic and cancer therapies. Inhibitors of P-gp have thus been advocated as promising candidates for overcoming the problem of MDR. However, due to lack of a high-resolution structure the concrete mode of interaction of both substrates and inhibitors is still not known. Therefore, structure-based design studies have to rely on protein homology models. In order to identify binding hypotheses for propafenone-type P-gp inhibitors, five different propafenone derivatives with known structure-activity relationship (SAR) pattern were docked into homology models of the apo and the nucleotide-bound conformation of the transporter. To circumvent the uncertainty of scoring functions, we exhaustively sampled the pose space and analyzed the poses by combining information retrieved from SAR studies with common scaffold clustering. The results suggest propafenone binding at the transmembrane helices 5, 6, 7 and 8 in both models, with the amino acid residue Y307 playing a crucial role. The identified binding site in the non-energized state is overlapping with, but not identical to, known binding areas of cyclic P-gp inhibitors and verapamil. These findings support the idea of several small binding sites forming one large binding cavity. Furthermore, the binding hypotheses for both catalytic states were analyzed and showed only small differences in their protein-ligand interaction fingerprints, which indicates only small movements of the ligand during the catalytic cycle.

## Author Summary

A major reason for the failure of cancer, antibiotic and antiviral therapies is the development of multidrug resistance (MDR). P-glycoprotein (P-gp), an ATP-dependent transport protein located in the membrane of epithelial cells of the kidney, liver, pancreas, colon and the blood-brain barrier, has been linked to the export of a broad variety of xenotoxins. Overexpression of P-gp leads to extrusion of therapeutic drugs and therefore triggers MDR. Thus, identification of potential P-gp inhibitors represents a promising concept for treatment of multiresistant tumours. However, due to lack of high resolution structural information and the polyspecific ligand recognition pattern only very limited information is available on the molecular basis of ligand/transporter interaction. Within this study we characterized the propafenone binding site of P-gp by docking a set of derivatives with known SAR into homology models of P-gp which represent both the apo and the nucleotide-bound state. Poses retrieved are in accordance with results from previous photoaffinity labeling studies and thus pave the way for structure-based *in silico* screening approaches.

## Introduction

The development of multidrug resistance (MDR) is one major impediment in cancer and antibiotic therapies [1]–[3]. In 1976 Juliano and Ling were able to associate the occurrence of MDR with the presence of P-glycoprotein (P-gp), the most prominent member of the adenosine triphosphate (ATP) binding cassette (ABC) transporter superfamily [4]–[6]. ABC proteins are energy dependent transporters with P-gp (ABCB1), multidrug resistance protein 1 (MRP1, ABCC1) and breast cancer resistance protein (BCRP, ABCG2) playing an important role in the protection of cells from harmful xenotoxins. Additionally, ABC proteins are known for modulating the pharmacokinetic profile of drugs and therefore the food and drug administration (FDA) suggested that new drug candidates should be routinely screened for P-gp interaction [7]. In this respect reliable *in silico* methods to characterize P-gp interaction would be of great benefit and help to render the drug discovery process more efficient [8]. However, the polyspecificity of the transporter poses a remarkable challenge concerning this task [9]. A number of ligand based studies have been conducted and provide some insights into the molecular basis of ligand/transporter interaction [10], [11]. With the help of biochemical studies like cysteine-cross linking, arginine scanning or photoaffinity labeling, amino acids contributing to binding of selected substrates were identified. On grounds of these experiments interaction sites for verapamil, rhodamine (R-site), Hoechst (H-site) and of cyclic peptide P-gp inhibitors (CPPI's) in the transmembrane (TM) domains (TMDs) of P-gp have been postulated [12]–[16]. Following the ABC transporter topology, P-gp possesses two TMDs, each consisting of 6 TM helices (TMHs), and two nucleotide binding domains (NBDs). While the TMDs are generally responsible for ligand interaction, ATP binding and hydrolysis takes place at the highly conserved nucleotide binding domains (NBDs) [17]. In case of propafenone type ligands photoaffinity labeling studies proposed two symmetrical binding regions at the interfaces of TMHs 5/8 and TMHs 2/11, respectively [18], [19]. Nevertheless, due to the small number and the low resolution of crystal structures of ABC-exporters, concrete binding hypotheses remain to be elucidated [20]. The lack of high resolution structures can be explained by the fact that ABC efflux pumps are located in the membrane and that they are rather flexible proteins. As energy dependent transporters they undergo large structural changes during one catalytic cycle, comprising ligand and ATP binding, ligand release and nucleotide hydrolysis [17], [21], [22]. Up to now the structure of human P-gp could not be resolved, for which reason homology models relying on bacterial homologues had to be utilized. With respect to this the bacterial transporters Sav1866 and MsbA structures, representing different catalytic states of the transport cycle, were generally used as templates [20]. In 2009 the crystal structure of mouse P-gp [12] in complex with a cyclic tetrapeptide was resolved, thus representing a ligand binding competent conformation of the protein. With 88% sequence identity it is well suited for homology modeling of the human homologue and thus paves the way for structure-based approaches.

**Figure 1:**
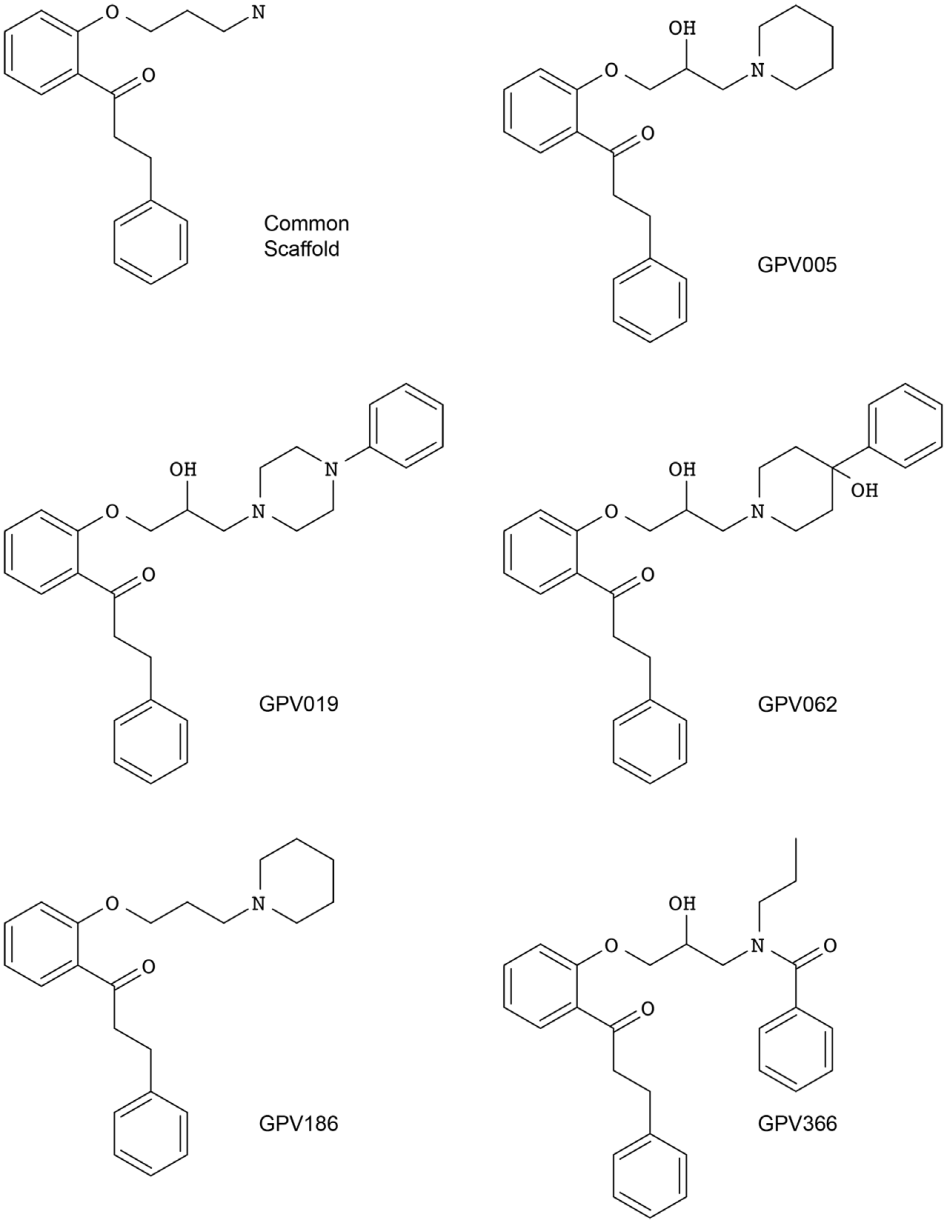
Ligand structures and codes that were used in this study. The common scaffold represents the largest common substructure and was used for root mean square deviation (RMSD) clustering.

The present study aimed at elucidating the binding mode of propafenone type inhibitors of P-gp using a combined homology modeling/docking approach. Propafenones show a clear structure-activity relationship (SAR) pattern [11] and thus represent versatile tool compounds to pursue this task. The wealth of ligand-based information available allows judging the reliability of docking poses on basis of the SAR pattern rather than by use of energetic terms derived from scoring functions. The selected compounds were the piperidine analogue GPV005, the analogous des-hydroxy derivative GPV186, the arylpiperazine GPV019, the hydroxyphenylpiperidine GPV062, and the benzoylamide GPV366 (Figure 1). All compounds bear a carbonyl group, which has been shown to be important for high P-gp inhibitory activity [23].

There are numerous studies showing that there is a basic underlying correlation between P-gp inhibitory activity and lipophilicity of the compounds. This accounts for several compound classes and has also been shown for propafenone analogues.

However, propafenones which bear a 4-hydroxy-4-phenylpiperidine moiety are generally by a factor of 10 more active than equi-lipophilic derivatives without the hydroxy-group in 4-position of the piperidine moiety (Figure S1) [24]. This points at a distinct additional interaction mediated by the 4-hydroxy group, most probably in form of a H-bond. This distinct SAR pattern in combination with the recently described common scaffold clustering [25], [26] was used to guide the prioritization of docking poses.

## Results

### Homology Modeling

In March 2009 Aller et al. published the crystal structure of mouse P-gp in the absence of a ligand (PDB ID: 3G5U) [12] and in complex with stereoisomeric CPPI's (PDB IDs: 3G60, 3G61) [12]. These structures represent the ligand binding competent state and were therefore the first choices for investigating drug/P-gp binding.

As the structural difference between the apo protein and the co-crystallized structures was surprisingly low (0.61 Å of Ca atoms) the higher resolved 3G5U structure was utilized as homology modeling template (3G5U_Pgp). With the modeling program MODELLER 100 different homology models were created and refined. All models were assessed with the geometry check tool implemented in MOE, which was used as a selection criterion for the final model. As additional measure for model quality the GA341 method was used, which relies on sequence identity, compactness and the combined statistical z-score. All models obtained the highest possible GA341 value of 1. Furthermore, the final model was analyzed with the structure assessment program PROCHECK [27]. The Ramachandran plot showed that 84.6% of the residues lie in most favored, 12.5% in additionally allowed, 2.1% in generously allowed and 0.8% in disallowed regions. The 2.9% of residues in generously allowed or disallowed regions are located in the nucleotide binding domains (NBD) or extracellular loops (ECL) and are therefore not involved in drug binding (Figure S2). The QMEAN analysis [28] (Figure S3) showed that residues lining the binding pocket are of satisfactory quality.

In order to cover different catalytic states of the protein, a second homology model was generated on basis of the bacterial transporter Sav1866 in the nucleotide-bound state (PDB code: 2HYD) [29] (2HYD_Pgp). This crystal structure is the highest resolution ABC exporter structure and has therefore been frequently used as modeling template [20]. 100 different models were generated and refined with MODELLER, of which all obtained a GA341 score of 1. The final model was selected on basis of the geometry check function in MOE. The Ramachandran plot statistics provided by the evaluation tool PROCHECK showed that 92% of all residues lie in most favored regions, while 6.5% were found in additionally allowed, 0.2% in generously allowed and only 0.6% in disallowed regions (Figure S2). Most of the 0.8% residues that are located in generously allowed or disallowed regions can be found in the NDB. Although residue Y116 lies within the TMDs and could therefore be involved in drug binding, this residue is oriented outside the cavity. A Ramachandran analysis performed by MolProbity and MOE detected no outliers in the TM region. Furthermore, this model shows also good quality in the binding site region according to QMEAN analysis (Figure S3).

### Docking

For the docking process five different propafenone derivatives were selected according to their differences in lipophilic efficiency and fit quality [30], and were docked into both homology models.

With the genetic algorithm based docking program GOLD [31] 100 poses for each of the five ligands were generated. To determine the ASN, GLN and HIS flips the web application MolProbity was utilized [32]. In order to avoid any bias, the binding site was defined as the complete TM region. According to the binding site assessment tool implemented in the software suite Schrödinger (SiteMap), this region in 3G5U_Pg mainly shows highly hydrophobic characteristics, which prompted us to dock the ligands in their non-ionized state. This is also supported by previous findings of ligand-based QSAR studies which indicated that the nitrogen atom not necessarily interacts in its charged form. However, since there is evidence that the protein's pore is water filled, the ligands were also docked in their ionized state [33]. This is also in accordance with recently published data which show that mutation of two glutamine residues at the entry path of the transporter to positively charged arginines affected the inhibitory activity of an positively ionizable propafenone analog, whereas the activity of GPV366 remained unmodulated [34].

**Figure 2:**
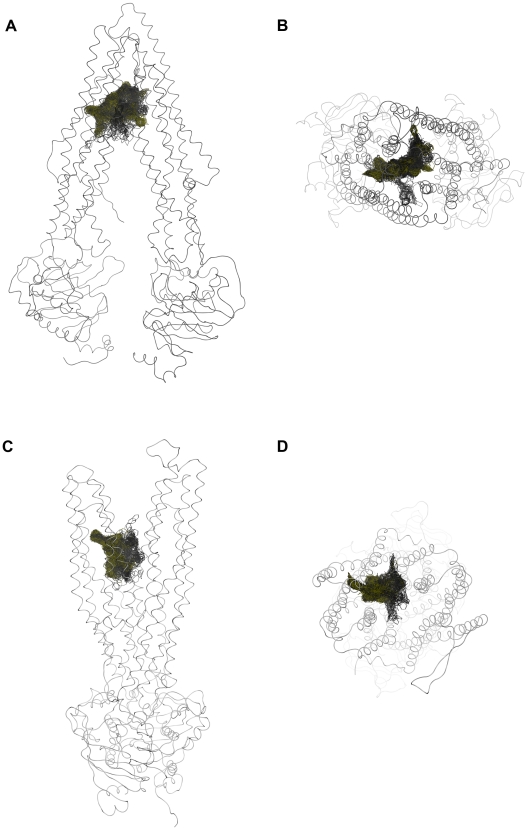
Distribution of all 500 poses in 3G5U_Pgp (A, B) and 2HYD_Pgp (C, D). Yellow: common scaffold cluster, grey: residual poses.

**Figure 3:**
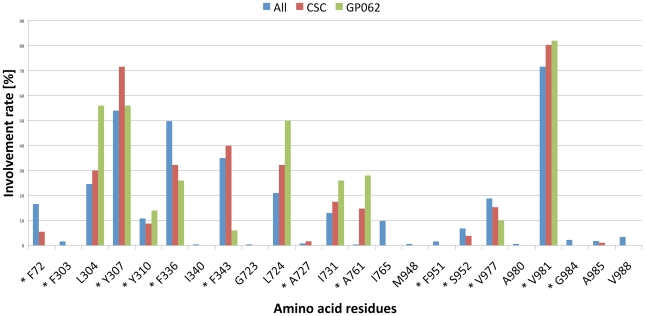
Protein ligand interaction fingerprint (PLIF) of the docking poses in 3G5U_Pgp, calculated with MOE. All: 500 poses after docking, CSC: common scaffold cluster, GPV062: cluster that showed an interaction between the OH-group of GPV062 and the protein. Residues marked with an asterisk show direct interaction with docking poses.

The resulting poses in both conformations were distributed largely within the TM region of P-gp (Figure 2), showing interactions with protein residues of multiple TM helices, located throughout the binding region. The calculation of protein ligand interaction fingerprints (PLIF) with MOE showed that in case of 3G5U_Pgp residues primarily located on TM helices 1, 5, 6, 7, 8, 11 and 12 were involved in binding (Figure 3). According to this tool, residues involved either show direct interactions with docking poses or are located within 4.5 Å distance to the ligand.

The unprocessed complexes were energetically minimized using LigX, a minimization tool implemented in MOE for further evaluation.

The minimized poses were clustered according to the root-mean-square deviation (RMSD) of the heavy atoms of the common scaffold (Figure 1) [35]. To follow the idea of a common binding mode only those clusters were kept that comprehend at least four out of the five compounds used (common scaffold clusters, CSCs). Clustering the poses of the docking run with 3G5U_Pgp resulted in 114 clusters, which were subsequently reduced to 12 CSC. As can be seen in Figures 2a and b some clusters protrude into the central cavity, but most of the CSCs are found in the vicinity of helices 5 and 8 (called the 5/8 interface). Previous photo-affinity labeling experiments suggested this region to be in involved in propafenone binding [36]. The position of the CSCs close to the 5/8 interface was also reflected in the PLIF pattern, as the involvement of residues L304 and Y307 located in TM helix 5, F343 of TM helix 6, L724 and I731 in TM helix 7, A761 in TM helix 8 and V981 in TM helix 12 was increased (Figure 3).

**Figure 4:**
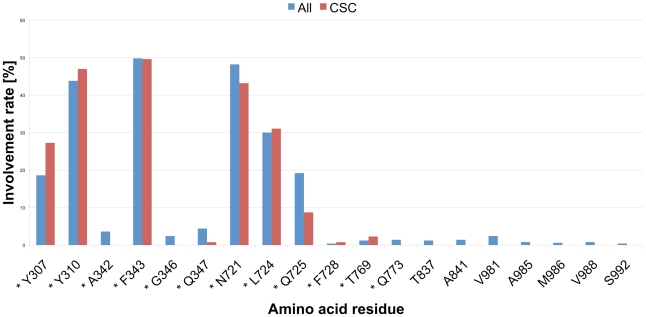
Protein ligand interaction fingerprint (PLIF) of the docking poses in 2HYD_Pgp, calculated with MOE. All: 500 poses after docking, CSC: common scaffold cluster. Residues marked with an asterisk show direct interaction with the docking poses.

In case of 2HYD_Pgp, the RMSD clustering process resulted in 78 clusters, which were reduced to nine common scaffold clusters, containing 264 poses (Table 1). As can be seen in Figure 2c and d, also docking into the nucleotide-bound homology model results in CSCs that tend to accumulate closer to the 5/8 interface and thus in vicinity of the photo-affinity labeled residues (Figure S4). The clustering process did not change the general PLIF pattern. TM helices 5, 6, 7 and 8 show similar contributions before and after scaffold clustering, but more frequently interactions were observed with individual residues, like Y307 (TM helix 5), Y310 (TM helix 5), L724 (TM helix 7) and T769 (TM helix 8) (Figure 4).

**Table 1:** Cluster statistics of docking runs into different catalytic states.

	3G5U_Pgp (non-ionized)	3G5U_Pgp (ionized)	2HYD_Pgp (non-ionized)	2HYD_Pgp (ionized)
*Total number of poses*	500	500	500	500
*Number of clusters after RMSD clustering (3 Å)*	114	111	78	77
*Number of common scaffold clusters (CSCs)*	12 (184 poses)	11 (195 poses)	9 (264 poses)	7 (240 poses)
*CSCs with interaction between GPV062-OH and protein*	I, II, III	IV, V, VI	−	−
*ph4-matching clusters*	I, III	IV, V, VI	−	−

The model based on the murine 3G5U structure represents the binding competent state, whereas the model based on the nucleotide-bound 2HYD structure likely represents the off-state of P-gp ligands [37]. Since propafenones might show different affinities towards these two structures, final pose evaluation was carried out in different ways.

In the hit-to-lead decision process as well as in lead optimization different efficiency metrics are applied to prioritise lead candidates. Briefly, in case of equi-potent compounds these parameters select for the smaller, more hydrophilic ones. As high lipophilicity correlates with promiscuity, poor solubility and poor metabolic clearance [38], candidates with high lipophilic efficiency (LLE  =  log(potency) - logP) are preferred. Ligand based studies clearly demonstrate a correlation between lipophilicity of P-gp inhibitors and their biological activity. However, as P-gp is extracting its ligands directly out of the membrane bilayer, this is most probably a consequence of concentration in the membrane rather than of direct protein interaction. Calculating the LLE normalizes for this effect and aids in identifying ligands with increased activity as a result of direct interaction with the protein rather than higher biomembrane distribution. The 4-hydroxy-4-phenylpiperidine GPV062 shows by far the highest LLE (Table 2) suggesting that in contrast to the other ligands, the higher activity of GPV062 is not due to a high logP value. While LLE normalizes for the lipophilic bias in potency description, LE simply corrects for the size of a molecule by dividing the activity of a compound by its heavy atom count. This approach is extensively used in fragment based drug design to select those fragments, which are worth being further investigated. As Reynolds et al. [30] concluded that LE generally is biased towards smaller molecules, the normalized size-independent fit quality (FQ) was assessed. Both, LE and FQ, clearly highlight the hydroxyphenylpiperidine GPV062 as being the most efficient compound (Table 2). The explanation for the increased LLE and FQ values seems to be the 4-hydroxy-group of GPV062. As this group clearly reduces the lipophilicity of a molecule, the increase in activity was interpreted as a result of hydrogen bonding. Thus, those CSCs were prioritized in which GPV062 is able to form a hydrogen bond between the hydroxyl-group of the 4-hydroxy-4-phenyl moiety and the protein.

**Table 2:** Activities of docked ligands.

Ligand	pIC50	HAC	ClogP	LLE	LE	FQ
*GPV005*	6.22	27	4.38	1.84	0.23	0.77
*GPV019*	6.21	33	5.15	1.06	0.19	0.75
*GPV062*	7.24	34	4.15	3.09	0.21	0.87
*GPV186*	6.19	26	5.54	0.65	0.24	0.77
*GPV366*	5.78	33	4.94	0.84	0.18	0.70

HAC  =  heavy atom count, LLE  =  lipophilic ligand efficiency, LE  =  ligand efficiency, FQ  =  fit quality.

**Figure 5:**
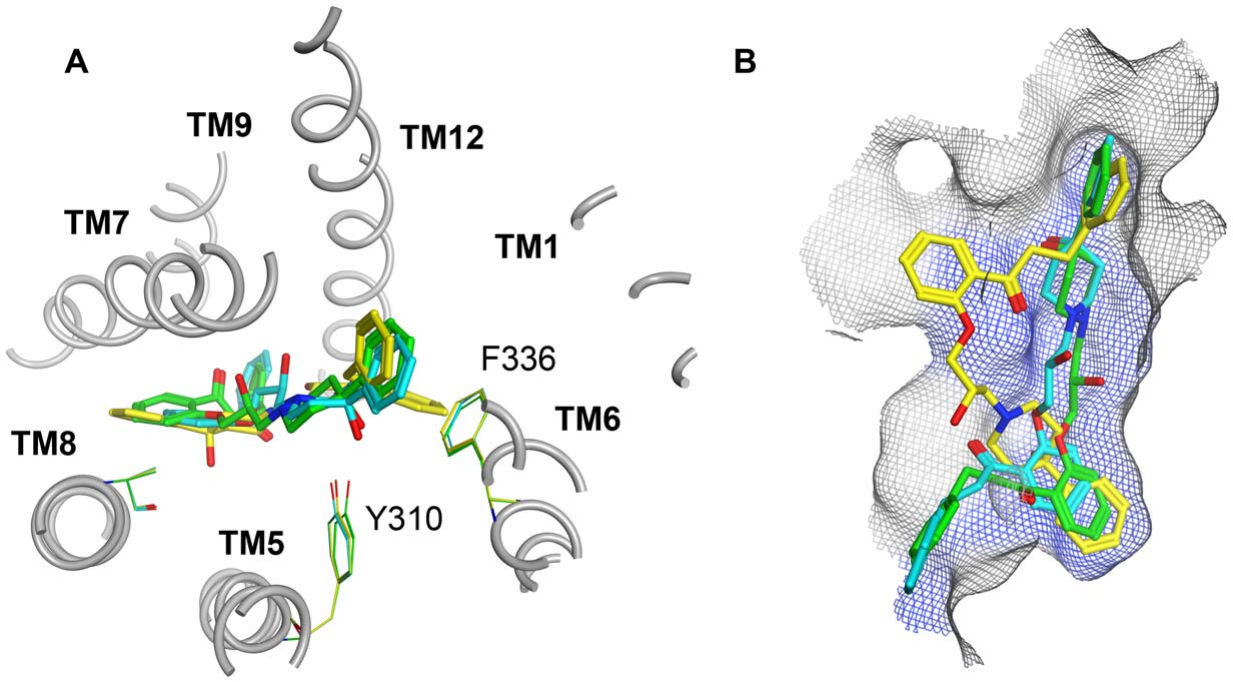
GPV062-OH interaction clusters in the binding pocket of 3G5U_Pgp. CSC I (green), CSC II (yellow), CSC III (cyan). A) Top view; the three interacting amino acids are colored according to their cluster-membership. B) side view; the blue surface indicates residues that are involved in propafenone binding, determined by photoaffinity labeling [14].

With 3G5U_Pgp only one quarter of all twelve common scaffold clusters showed a hydrogen bond between the hydroxyl-group of GPV062 and the protein (Table 1) (GPV062-OH Clusters). These three clusters (CSCs I, II, III) are located very close to each other at the 5/8 interface (Figure 5a), with an increased number of interactions formed by residues L304, Y310, L724, A761 and V981. Furthermore, the PLIF pattern showed that interactions with TM helices 1 and 11 are no longer present.

The positions of CSCs I and III are very similar, since both are forming a hydrogen bond with Y310 and a π/π-interaction with F336. In CSC II, on the contrary, a hydrogen bond interaction with A761 was observed.

For further evaluation of the poses a pharmacophore search was performed, utilizing a model published by Langer et al. that based on a set of propafenone type P-gp inhibitors [39]. Only those two clusters that formed a hydrogen bond with Y310 matched this pharmacophore query. As depicted in Figure 5b, both clusters perfectly fit the photolabeling pattern observed in this half of the protein.

**Figure 6:**
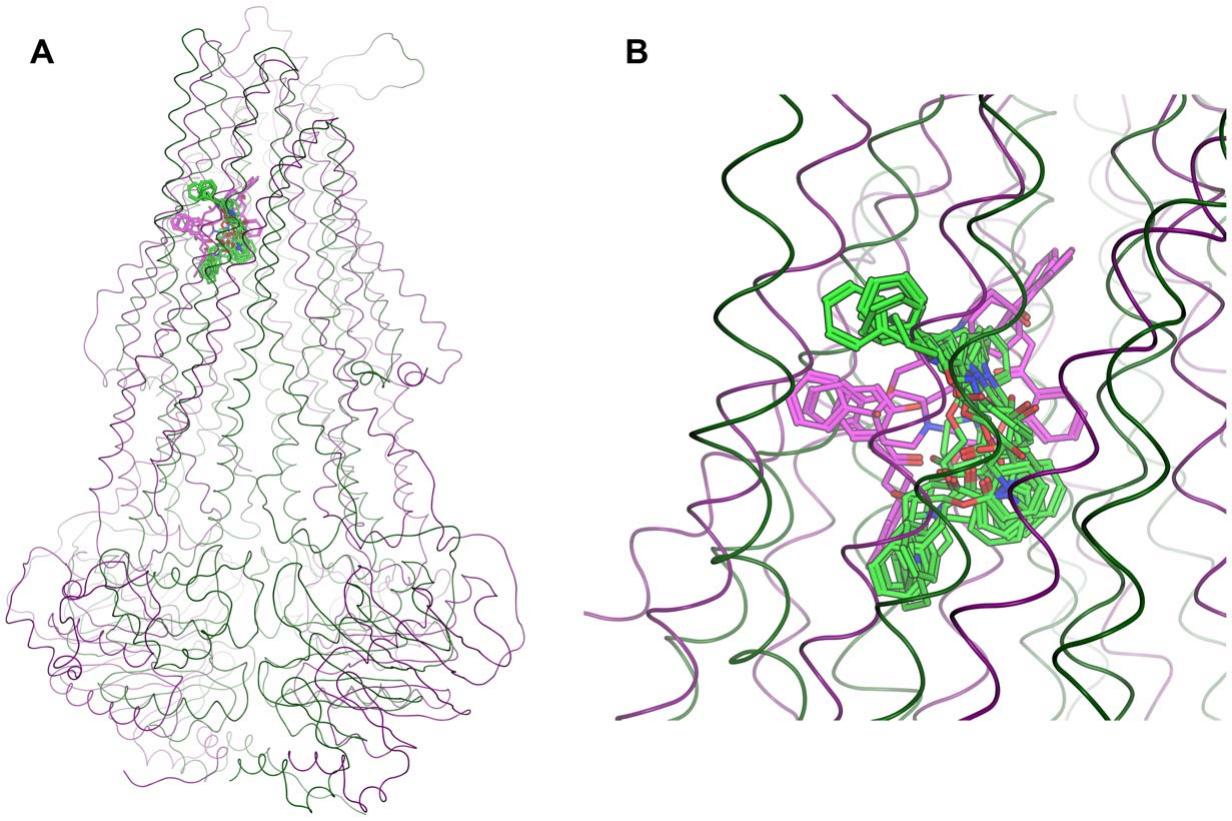
Comparison of docking poses in different stages of the catalytic cycle. Magenta: GPV062-OH clusters of docking into 3G5U_Pgp (high affinity), green: common scaffold clusters of docking into 2HYD_Pgp (low affinity). A) overview, B) close-up view.

Evaluation of the docking results with 2HYD_Pgp could not be based on ligand affinity data, since this structure represents the nucleotide-bound off-state and therefore is considered as the low-affinity state for substrates. This rules out prioritization on basis of SAR-information. All common scaffold clusters of 2HYD_Pgp are in close vicinity of the 3G5U_Pgp GPV062-OH poses (Figure 6).

## Discussion

### Homology Modeling

The homology models generated in this study resemble two different states of P-gp: the open-inward or apo state and the open-outward or nucleotide-bound state. Since the publication of mouse P-gp in the absence (PDB ID: 3G5U) or in complex with ligands (PDB Ids: 3G60, 3G61) only a few homology models of the human homologue were published on the basis of these structures. Pajeva et al. presented two homology models that were based on the structure of 3G61, chain A, which is complexed with QZ59-SSS [40], [41]. The advantage of selecting this template for homology modeling is the presence of the complexed ligands. On the other hand, 3G5U is resolved at higher resolution (3.80 Å) and shows only minor differences in the binding site (RMSD of all atoms of QZ59-SSS surrounding residues: 1.251 Å). However, the still relatively low resolution of the template certainly needs to be taken into account when it is used for docking experiments.

The open-outward model relied on the structure of the bacterial homologue Sav1866 (PDB ID: 2HYD), which possesses the same domain architecture as P-gp [42] and therefore frequently served as modeling template. With a resolution of 3.0 Å it represents one of the best resolved full ABC transporters. The relevance of this nucleotide-bound structure is widely accepted, as experimental studies showed close association of the NBDs [43], [44]. In contrast, the structures of mouse P-gp disagree with kinetic and FRET studies that report no complete dissociation of the NBDs [37], [45]. In addition, a recent cross-linking study further strengthened this by showing that an M1M cross-link between L175C and N820C did not prevent verapamil and rhodamine B to be transported [46]. However, as P-gp is known to be highly flexible and to undergo large conformational changes during the catalytic cycle, the existence of a state with dissociated NBDs cannot be ruled out entirely. Additional evidence was presented by Sauna et al., who demonstrated that ATP binding reduces the affinity for propafenone analogues [37]. Finally, the fact that the mouse P-gp structure (3G5U) has been cocrystallized with two ligands strongly indicates that this structure represents a ligand-binding competent state of the protein. Thus it was considered as a versatile template for modeling the high-affinity state of the protein for subsequent docking studies.

### Docking

Although ligand docking is a commonly used tool for the identification of ligand-protein interactions, in case of P-gp it bears a lot of challenges: (i) P-gp possesses a large binding cavity that consists of several binding sites, (ii) is highly flexible, and (iii) is probably able to harbor more than one ligand simultaneously [47], [48]. Finally, there is no high resolution structure of human P-gp available, which requires to work with protein homology models. Considering the low resolution of the templates, this adds additional layers of uncertainty. Thus, results from ligand docking runs have to be interpreted very carefully. In an attempt to combat all these uncertainties we applied an exhaustive docking protocol avoiding to a maximum possible extent the use of scoring functions and including all the knowledge present from SAR and QSAR studies.

In docking experiments, the definition of the binding site is a key parameter of the docking protocol. As only little information is available about binding of propafenones into P-gp, the whole TM region was selected as a potential interaction region. In order to avoid any bias introduced by scoring functions, a large amount of docking poses was generated. While placement algorithms of docking programs are most of the time able to find the native pose of a ligand in the binding pocket, the correct estimation of the binding energy leading to a correct ranking of the poses is still unsatisfying. To overcome this uncertainty of scoring functions, we recently implemented experimental data guided docking/scoring. In this approach prioritization of docking poses is performed on basis of mutagenesis data, biochemical data, and/or information from ligand based studies [25], [26].

The interaction of propafenones with P-gp follows a clear structure-activity relationship pattern (for reviews see [11]). Based on these results and on calculation of lipophilic efficiency (LLE) and fit quality (FQ) we selected a small set of analogs for docking and subsequent common scaffold clustering. Both LLE and FQ as well as previously performed Hansch analysis stressed the importance of the hydroxyl-group of GPV062 for high activity. Clustering of all poses according to their common scaffold (Figure 1) combined with pose selection based on H-bonding interactions of the OH group allowed a considerable reduction of docking poses.

**Figure 7:**
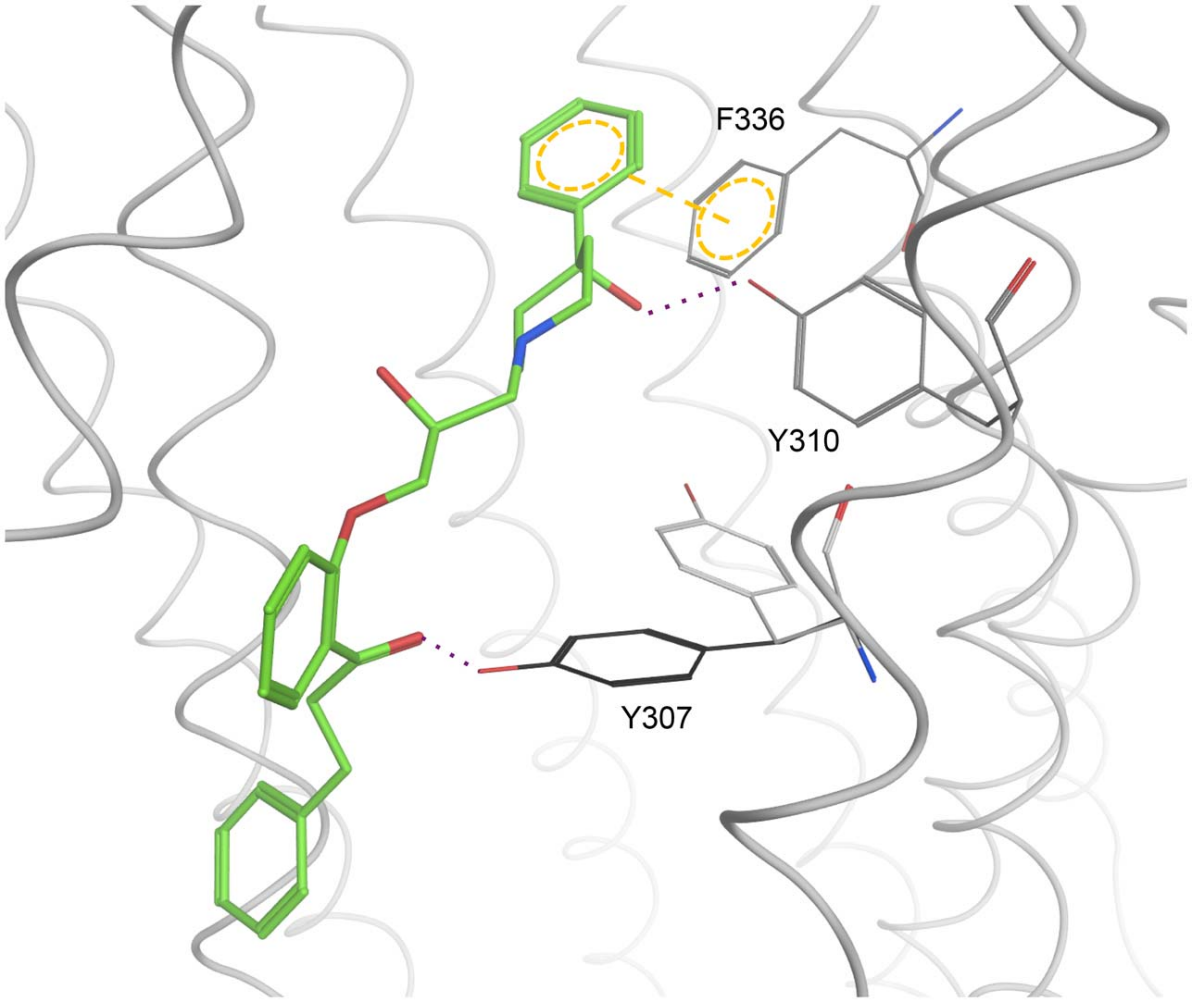
Interactions of CSC I with 3G5U_Pgp. By rotating the residue Y307 (grey:original, black: rotated) a new hydrogen bond between Y307 and the carbonyl group of the ligand was formed.

Although docking experiments have their limitations depending on the validity of the target structure, the results of docking into 3G5U_Pgp are very consistent. As shown in Figure 5 the three final clusters are located in close vicinity. Especially CSCs I and III are very similar, showing strong H-bonding interactions with Y310 and thus supporting the importance of the hydroxyl group of GPV062. Both clusters also match the pharmacophore model of Langer et. al [39]. Due to previously performed ligand based studies also the importance of the carbonyl group of the propafenone scaffold became evident [49]. Although initial poses show no interaction with the carbonyl group, these become apparent after processing of data with the rotamer explorer implemented in MOE. When rotating amino acid residue Y307 towards the carbonyl group, an interaction can be generated (Figure 7). In a dynamic system H-bond formation thus might be observed. Interestingly, for CSC III a rotation of Y307 did not result in an interaction with the carbonyl group, most probably due to a small offset of the carbonyl group towards the cell interior. However, this assumption would need further investigations, since discussing possible interactions on atomistic detail has to be done with caution when working with a homology model, especially if the resolution is quite low. Nevertheless, the relevance of Y307 in ligand binding was also shown with cocrystallized CPPI's, where the R-stereoisomer forms an interaction with this residue [12]. Furthermore, this residue is in close vicinity to I306, which was shown to lead to permanent activation of ATPase activity when mutated to cysteine and covalently linked with the thiol-reactive drug substrate verapamil [15].

CSC II forms a weak H-bond between the hydroxyl-group of GPV062 and the backbone of A761. With respect to the ligand interaction tool in MOE the strength of this bond is only 1/10 compared to that in CSCs I and III. Applying the rotamer explorer results in either formation of a stronger hydrogen bond with the OH-group of GPV062 or formation of a new interaction with the carbonyl group (with these interactions not being coexistent). Finally, with respect to residues photoaffinity labelled by benzophenone analogous propafenones, CSCs I and III show a better match (Figure 5b), because the photoreactive carbonyl group is closer to the PAL region than in CSC I.

In consideration of these findings the pose of CSC I was preferred over the other two clusters.

It is also known that binding of propafenones to P-gp meets steric constraints in the vicinity of the nitrogen atom, because diphenyl moieties in this position lead to a log order decrease in activity [49]. In all three clusters the introduction of a diphenyl substituted nitrogen results in steric clashes and subsequent minimization of the binding pocket leads to the loss of H-bond interactions.

Docking into 3G5U_Pgp with ionized ligands resulted in three different CSCs that show an interaction between the OH_group of GPV062 and the protein. While one is located very central in the pore (CSC IV) forming an H-bond between GP062-OH and A727, the other two (CSC V and VI) exactly match CSC I of the docking with neutral ligands. For the latter an H-bond between the hydroxyl-group and Y310 could be observed.

As can be seen in Figure 2, the different CSCs of 2HYD_Pgp are located in the same binding site at the 5/8 interface. Regarding their different orientation within this region, docking poses can be separated into two distinct groups. Docking poses belonging to group 1 (CSCs a, b, c and d) frequently form interactions between the carbonyl group and Y307. Furthermore, H-bond interactions between the piperidine nitrogen or the hydroxyl-group and Y310 can be observed. This interaction pattern is similar to the one of CSCs I and III of the docking run performed with 3G5U_Pgp. Individual GPV062 poses show additional H-bond interactions between the 4-hydroxy-group and Y310, another frequently observed interaction in CSCs I and III. According to these observations the transformation of CSCs I and III in the apo state into CSCs of group 1 of the nucleotide-bound state seems possible.

In contrast, group 1 and group 2 are in an up-side-down orientation when compared to each other. In this case the carbonyl group is located near Y310 and thus closer to the extracellular portion of the protein. The nitrogen atom, as well as the hydroxyl group, is oriented towards Y307 and N721, which was also observed for CSC II of the 3G5U_Pgp docking run. Therefore, group 2, comprising clusters e, f and g, corresponds to the nucleotide-bound conformations of CSC II of the apo-conformation.

CSCs h and i cannot be clearly assigned to one of these groups and have to be regarded separately. The nitrogen atom of CSC h shows a similar location as the N of group 2, however, due to a shift of the central phenyl ring downwards, H-bond interactions between the carbonyl oxygen and Y307 and the OH-group and N721 can be formed simultaneously.

CSC i shares its carbonyl group orientation with group 2, but the central phenyl ring lies in a perpendicular direction, which results in interactions between the ligand nitrogen and hydroxyl group with Q725.

Considering the docking run to 2HYD_Pgp with ionized ligands, group 1 could be clearly reproduced. Three out of seven CSCs form those characteristic H-bond interactions between the carbonyl oxygen and Y307 and the hydroxyl group and Y310. In contrast to the unprotonated ligands, the nitrogen atom and Y310 form a pi/cation interaction and occur at higher frequency. Overall the clusters belonging to group 1 show high homogeneity and strong interactions. In contrast to this the poses of each of the four other clusters share no consistent pattern and therefore the common binding was only reflected in geometrically similar positioning.

Interestingly, although the experimental data suggest two symmetrical binding sites, no common scaffold cluster and hardly any poses could be found at the second photoaffinity labeled site at the 2/11 interface. One possible explanation might be the asymmetry of the template crystal structure 3G5U. The region consisting of TM helices 4, 5, 7, 8, 9 and 12 in case of 3G5U_P-gp, and TM helices 3, 4, 5, 6, 7 and 8 in case of 2HYD_P-gp, in both cases showed larger sites when using the SiteFinder tool in MOE than their counterparts around the 2/11 interface. This demonstrates the limitations of docking experiments relying on one crystal structure that represents only a snapshot of a flexible protein. Thus, to rule out the possibility that every docked ligand will end up at the 5/8 interface just because of this asymmetry, a docking run with rhodamine 123 was conducted. In this case 21 of 39 clusters were found in vicinity of residues I340, L975 and V981, which are located on TM helices 6 and 12 and known to be involved in rhodamine binding [13].

### Comparison of Open-Closed Binding Regions

In order to gain first insights into the potential ligand translocation pathways, the compounds were docked in two different catalytic states of P-gp. Interestingly, the docking results show similar interaction patterns. In both models, ligand poses are found in close vicinity (4,5 Å) of residues Y307 and Y310 of TM helix 5, F343 of TM helix 6 and L724 of TM helix 7, which suggests involvement of both TM domains in drug binding. This is in accordance with Loo et al., who showed that both TM domains are essential for drug translocation [50].

**Figure 8:**
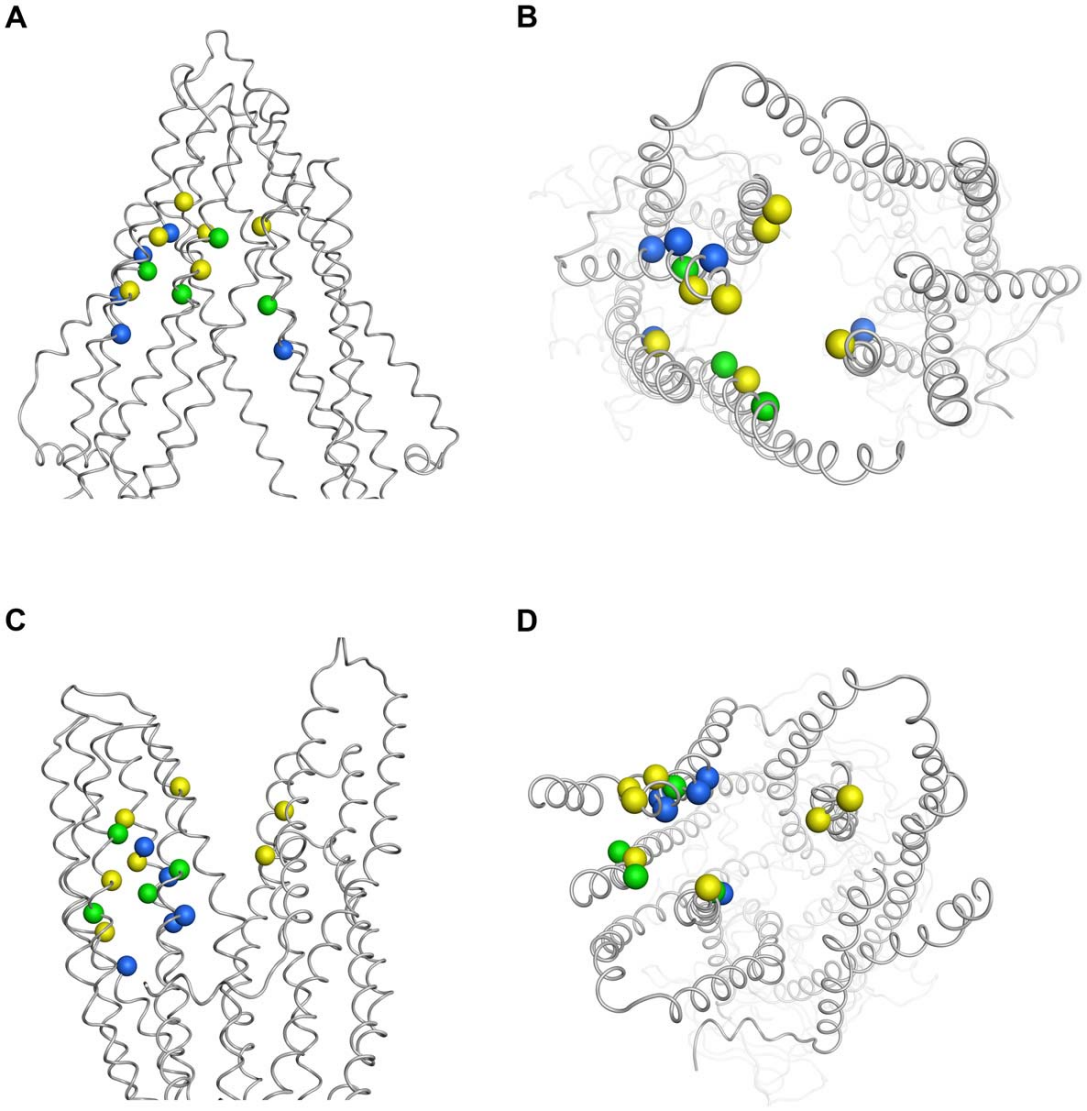
Comparison of main interacting residues. The spheres represent Cα-atoms of interacting residues of 3G5U_Pgp (panels A, B) and 2HYD_Pgp (panels C, D). Blue spheres: 2HYD_Pgp, green: 2HYD_Pgp and 3G5U_Pgp, yellow: 3G5U_Pgp. A) and b) 3G5U_Pgp in front and top view; c) and d) 2HYD_Pgp in front and top and view.

In Figure 8 the interacting amino acid residues of both docking approaches are depicted. In the 3G5U_Pgp structure the interactions seem to be very similar, concentrating on the 5/8 interface. Due to the conformational change and the resulting movement of TM helix 12, interactions between propafenones and V977 and V981 are lost. Top views of the models indicate that the corresponding interacting residues (3G5U_Pgp: yellow, 2HYD_Pgp: blue, both: green) face the central pore. It seems that the conformational change associated with nucleotide binding moves previously buried residues towards the binding pocket and therefore allows them to form new interactions with the ligands.

**Figure 9:**
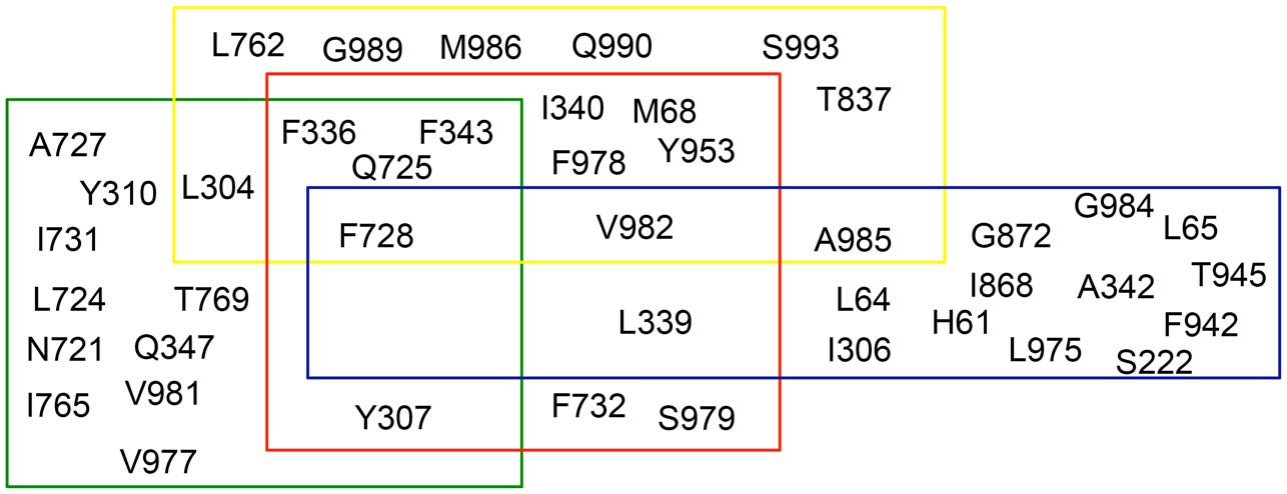
Venn diagram of drug binding sites in human P-gp.

In Figure 9 a Venn diagram compares residues in binding sites of CPPIs and verapamil with that of propafenones. As TM helices 5, 6 and 7 are lining the central cavity in the murine P-gp structure, a considerable overlap of residues interacting with propafenones and that shown to interact with cocrystallized CPPIs can be found in this region. One residue of TM helix 7, F728, is suggested to interact with all four drugs and therefore plays a crucial role in ligand binding. This is in agreement with the finding of Loo et al. that TM helix 7 is part of the drug binding site [51]. Loo et al. also demonstrated that binding of vinblastine, cyclosporin A and rhodamine B could prevent the formation of a cross-link between L339C and F728C, suggesting that the ligands are at least partially located between these two residues [52]. This is also the case for the three docking clusters in 3G5U_Pgp, which are presented in this study.

Furthermore, the diagram is consistent with the notion that P-gp possesses a large binding cavity, which harbors different partially overlapping drug binding sites for different ligands [39], [40]. In the cocrystallized structures 3G60 and 3G61 the cyclopeptides are located at the interface of the two TMDs, which explains the high overlap between these ligands and verapamil or propafenones, respectively.

Ligand docking into polyspecific antitargets such as the hERG potassium channel and the drug transporter P-glycoprotein requires thorough validation of the poses obtained. In this paper we describe the application of an SAR-guided docking protocol, which for the first time retrieves a binding hypothesis for propafenone-type inhibitors of P-gp. Although performing docking studies with homology models always bears a lot of risks the results are in agreement with experimental studies, which strengthens the applicability of the complex docking protocol we used for this study. This could pave the way for structure-based ligand design approaches.

## Methods

### Homology Modeling

Two homology models based on the bacterial homologue Sav1866 (PDB ID: 2HYD, resolution: 3.0 Å [29]) and murine P-gp (PDB ID: 3G5U, chain A, resolution: 3.8 Å [12]) were built. Both models were generated with the program MODELLER 9v7 using the automodel protocol [53]. In case of 3G5U_Pgp the alignment proposed by Aller et al. [8] was used (Figure S5). To correct the disruption in TM helix 12 of 3G5U a secondary structure constraint between residues 885 and 928 was applied. For 2HYD_Pgp the alignment was done according to Stockner et al. [54] (Figure S6). The linker region between the TM domains was modeled. Out of the 100 generated models those with the smallest number of outliers according to the geometry check function in MOE were selected for docking.

### Docking

For the docking study five propafenone derivatives were selected on basis of known SAR and differences in LLE and FQ. LLE was calculated by subtracting ClogP from experimentally determined IC_50_ values and FQ was calculated as outlined in [30]. To examine the quality of the ClogP calculation, the values were compared with previously published experimentally defined logP data of propafenone analogs [23]. A correlation of *r* = 0.92 could be identified.

Minimization and protonation of the ligands was performed with MOE.

For the correct determination of ASN/GLN/HIS flips the web application MolProbity was utilized [32]. The docking process was performed using the Gold Suite 1.2.1 [31]. Hydrogens were added and the binding site was defined as the entire TM region of the homology model. All side chains were kept rigid and the ligand was treated flexible by performing 100 genetic algorithm runs per molecule. The implemented Gold scoring function GoldScore was used for evaluation of the complexes. The final poses and the surrounding protein amino acid residues were minimized using LigX implemented in the MOE software package. Rescoring was performed with the empirical scoring function XSCORE.

### Cluster Analysis

On basis of the common scaffold an RMSD matrix of all five ligands was generated and used for clustering. The dissimilarity matrix was clustered with the program R [55], using complete linkage as clustering algorithm and a clustering height of 3 Å. Only those clusters were kept that inherited at least four out of the five ligands docked.

In case of 3G5U_Pgp those clusters were selected for final assessment that were able to form a hydrogen bond between the OH-group of GPV062 and the protein, detected by the ligand interaction tool of MOE.

## Supporting Information

Figure S1 **ClogP-pIC50 correlation of propafenone analogs.** The ligands used for docking are highlighted. [24]
(TIF)

Figure S2 **Outliers defined by PROCHECK analysis.** A) 3G5U_Pgp, B) 2HYD_Pgp. Grey: generously allowed residues, black: disallowed residues.
(TIFF)

Figure S3 **QMEAN analysis of the homology models generated with MODELLER.** A) 3G5U_Pgp, B) 2HYD_Pgp. Blue: high quality regions, red: low quality regions.
(TIFF)

Figure S4 **Common scaffold clusters after docking into 2HYD_Pgp.** The blue surface indicates residues that are involved in propafenone binding, determined by photoaffinity labeling [14].
(TIFF)

Figure S5 **Sequence alignment used for the generation of the homology model 3G5U_Pgp.** The sequences of human P-gp and of the X-ray structure of mouse P-gp have been aligned as suggested by Aller et al. [12].
(PDF)

Figure S6 **Sequence alignment used for the generation of the homology model of 2HYD_Pgp.** The sequences of human P-gp and the bacterial ABC-exporter SAV1866 have been aligned as suggested by Stockner et al. [54].
(PDF)

## References

[1] Dean M, Fojo T, Bates S (2005). Tumour stem cells and drug resistance.. Nat Rev Cancer.

[2] Globisch C, Pajeva IK, Wiese M (2008). Identification of putative binding sites of P-glycoprotein based on its homology model.. ChemMedChem.

[3] Ramachandra M, Ambudkar SV, Gottesman MM, Pastan I, Hrycyna CA (1996). Functional characterization of a glycine 185-to-valine substitution in human P-glycoprotein by using a vaccinia-based transient expression system.. Mol Biol Cell.

[4] Juliano R (1976). Drug-resistant mutants of Chinese hamster ovary cells possess an altered cell surface carbohydrate component.. J Supramol Struct.

[5] Hrycyna CA (2001). Molecular genetic analysis and biochemical characterization of mammalian P-glycoproteins involved in multidrug resistance.. Semin Cell Dev Biol.

[6] Ford RM, Kamis AB, Kerr ID, Callaghan R, Ecker G, Chiba P (2009). The ABC Transporters: Strucural Insights into Drug Transport.. Transporters as Drug Carriers.

[7] Food and Drug Administration (FDA) (2006). Drug Interaction Studies - Study Design, Data Analysis, and Implications for Dosing and Labeling.. http://www.fda.gov/downloads/Drugs/GuidanceComplianceRegulatoryInformation/Guidances/ucm072101.pdf.

[8] Schneider G Virtual screening: an endless staircase?. Nat Rev Drug Discov.

[9] Klepsch F, Stockner T, Erker T, Muller M, Chiba P (2010). Using structural and mechanistic information to design novel inhibitors/substrates of P-glycoprotein.. Curr Top Med Chem.

[10] Raub TJ (2006). P-glycoprotein recognition of substrates and circumvention through rational drug design.. Mol Pharm.

[11] Pleban K, Ecker GF (2005). Inhibitors of p-glycoprotein–lead identification and optimisation.. Mini Rev Med Chem.

[12] Aller SG, Yu J, Ward A, Weng Y, Chittaboina S (2009). Structure of P-glycoprotein reveals a molecular basis for poly-specific drug binding.. Science.

[13] Loo TW, Clarke DM (2002). Location of the rhodamine-binding site in the human multidrug resistance P-glycoprotein.. J Biol Chem.

[14] Loo TW, Clarke DM (1997). Identification of residues in the drug-binding site of human P-glycoprotein using a thiol-reactive substrate.. J Biol Chem.

[15] Loo TW, Clarke DM (2008). Mutational analysis of ABC proteins.. Arch Biochem Biophys.

[16] Qu Q, Sharom FJ (2002). Proximity of bound Hoechst 33342 to the ATPase catalytic sites places the drug binding site of P-glycoprotein within the cytoplasmic membrane leaflet.. Biochemistry.

[17] Linton KJ, Higgins CF (2007). Structure and function of ABC transporters: the ATP switch provides flexible control.. Pflugers Arch.

[18] Pleban K, Kopp S, Csaszar E, Peer M, Hrebicek T (2005). P-glycoprotein substrate binding domains are located at the transmembrane domain/transmembrane domain interfaces: a combined photoaffinity labeling-protein homology modeling approach.. Mol Pharmacol.

[19] Chiba P, Mihalek I, Ecker GF, Kopp S, Lichtarge O (2006). Role of transmembrane domain/transmembrane domain interfaces of P-glycoprotein (ABCB1) in solute transport. Convergent information from photoaffinity labeling, site directed mutagenesis and in silico importance prediction.. Curr Med Chem.

[20] Klepsch F, Ecker G (2010). Impact of the Recent Mouse P-Glycoprotein Structure for Structure-Based Ligand Design.. Mol Inf.

[21] Seeger MA, van Veen HW (2009). Molecular basis of multidrug transport by ABC transporters.. Biochim Biophys Acta.

[22] Callaghan R, Ford RC, Kerr ID (2006). The translocation mechanism of P-glycoprotein.. FEBS Lett.

[23] Chiba P, Ecker G, Schmid D, Drach J, Tell B (1996). Structural requirements for activity of propafenone-type modulators in P-glycoprotein-mediated multidrug resistance.. Mol Pharmacol.

[24] Chiba P, Hitzler M, Richter E, Huber M, Tmej C (1997). Studies on Propafenone-type Modulators of Multidrug Resistance III: Variations on the Nitrogen.. Quant Struct-Act Relat.

[25] Sarker S, Weissensteiner R, Steiner I, Sitte HH, Ecker GF (2010). The high-affinity binding site for tricyclic antidepressants resides in the outer vestibule of the serotonin transporter.. Mol Pharmacol.

[26] Richter L, Ernst M, Sieghart W, Ecker G (2010). Identification of binding modes of benzodiazepeine binding site ligands by a combined docking-pharmacophore modeling approach.. Drugs Future.

[27] Laskowski RA, MacArthur MW, Moss DS, Thornton JM (1993). PROCHECK: A program to check the stereochemical quality of protein structures.. J Appl Crystallogr.

[28] Benkert P, Kunzli M, Schwede T (2009). QMEAN server for protein model quality estimation.. Nucleic Acids Res.

[29] Dawson RJ, Locher KP (2006). Structure of a bacterial multidrug ABC transporter.. Nature.

[30] Reynolds CH, Bembenek SD, Tounge BA (2007). The role of molecular size in ligand efficiency.. Bioorg Med Chem Lett.

[31] Verdonk ML, Cole JC, Hartshorn MJ, Murray CW, Taylor RD (2003). Improved protein-ligand docking using GOLD.. Proteins.

[32] Chen VB, Arendall WB, Headd JJ, Keedy DA, Immormino RM (2010). MolProbity: all-atom structure validation for macromolecular crystallography.. Acta Crystallogr D Biol Crystallogr.

[33] Gutmann DA, Ward A, Urbatsch IL, Chang G, van Veen HW (2010). Understanding polyspecificity of multidrug ABC transporters: closing in on the gaps in ABCB1.. Trends Biochem Sci.

[34] Parveen Z, Stockner T, Bentele C, Pferschy S, Kraupp M (2011). Molecular Dissection of Dual Pseudosymmetric Solute Translocation Pathways in Human P-Glycoprotein.. Mol Pharmacol.

[35] Chema D, Eren D, Yayon A, Goldblum A, Zaliani A (2004). Identifying the binding mode of a molecular scaffold.. J Comput Aided Mol Des.

[36] Ecker GF, Csaszar E, Kopp S, Plagens B, Holzer W (2002). Identification of ligand-binding regions of P-glycoprotein by activated-pharmacophore photoaffinity labeling and matrix-assisted laser desorption/ionization-time-of-flight mass spectrometry.. Mol Pharmacol.

[37] Sauna ZE, Kim IW, Nandigama K, Kopp S, Chiba P (2007). Catalytic cycle of ATP hydrolysis by P-glycoprotein: evidence for formation of the E.S reaction intermediate with ATP-gamma-S, a nonhydrolyzable analogue of ATP.. Biochemistry.

[38] Leeson PD, Springthorpe B (2007). The influence of drug-like concepts on decision-making in medicinal chemistry.. Nat Rev Drug Discov.

[39] Langer T, Eder M, Hoffmann RD, Chiba P, Ecker GF (2004). Lead identification for modulators of multidrug resistance based on in silico screening with a pharmacophoric feature model.. Arch Pharm (Weinheim).

[40] Pajeva IK, Globisch C, Wiese M (2009). Combined pharmacophore modeling, docking, and 3D QSAR studies of ABCB1 and ABCC1 transporter inhibitors.. Chem Med Chem.

[41] Pajeva IK, Globisch C, Wiese M (2009). Comparison of the inward- and outward-open homology models and ligand binding of human P-glycoprotein.. FEBS J.

[42] Zolnerciks JK, Wooding C, Linton KJ (2007). Evidence for a Sav1866-like architecture for the human multidrug transporter P-glycoprotein.. FASEB J.

[43] Lee JY, Urbatsch IL, Senior AE, Wilkens S (2008). Nucleotide-induced structural changes in P-glycoprotein observed by electron microscopy.. J Biol Chem.

[44] Loo TW, Bartlett MC, Clarke DM (2002). The “LSGGQ” motif in each nucleotide-binding domain of human P-glycoprotein is adjacent to the opposing walker A sequence.. J Biol Chem.

[45] Qu Q, Sharom FJ (2001). FRET analysis indicates that the two ATPase active sites of the P-glycoprotein multidrug transporter are closely associated.. Biochemistry.

[46] Loo TW, Bartlett MC, Clarke DM (2010). Human P-glycoprotein is active when the two halves are clamped together in the closed conformation.. Biochem Biophys Res Commun.

[47] Loo TW, Bartlett MC, Clarke DM (2003). Simultaneous binding of two different drugs in the binding pocket of the human multidrug resistance P-glycoprotein.. J Biol Chem.

[48] Lugo MR, Sharom FJ (2005). Interaction of LDS-751 and rhodamine 123 with P-glycoprotein: evidence for simultaneous binding of both drugs.. Biochemistry.

[49] Ecker G, Chiba P, Ecker G, Chiba P (2009). QSAR Studies on ABC Transporter - How to Deal with Polypspecificity.. Transporters as Drug Carriers.

[50] Loo TW, Clarke DM (1998). Superfolding of the partially unfolded core-glycosylated intermediate of human P-glycoprotein into the mature enzyme is promoted by substrate-induced transmembrane domain interactions.. J Biol Chem.

[51] Loo TW, Bartlett MC, Clarke DM (2006). Transmembrane segment 7 of human P-glycoprotein forms part of the drug-binding pocket.. Biochem J.

[52] Loo TW, Bartlett MC, Clarke DM (2007). Suppressor mutations in the transmembrane segments of P-glycoprotein promote maturation of processing mutants and disrupt a subset of drug-binding sites.. J Biol Chem.

[53] Sali A, Blundell TL (1993). Comparative protein modelling by satisfaction of spatial restraints.. J Mol Biol.

[54] Stockner T, de Vries SJ, Bonvin AM, Ecker GF, Chiba P (2009). Data-driven homology modelling of P-glycoprotein in the ATP-bound state indicates flexibility of the transmembrane domains.. FEBS J.

[55] R Development Core Team (2010). R: A Language and Environment for Statistical Computing.. http://www.R-project.org.

