# Supplementary figures and images for: Exhaustive Sampling of Docking Poses Reveals Binding Hypotheses for Propafenone Type Inhibitors of P-Glycoprotein
Source: PLoS Comput Biol. 2011 May 12;7(5):e1002036. doi: 10.1371/journal.pcbi.1002036 (PMC3093348; doi:10.1371/journal.pcbi.1002036)

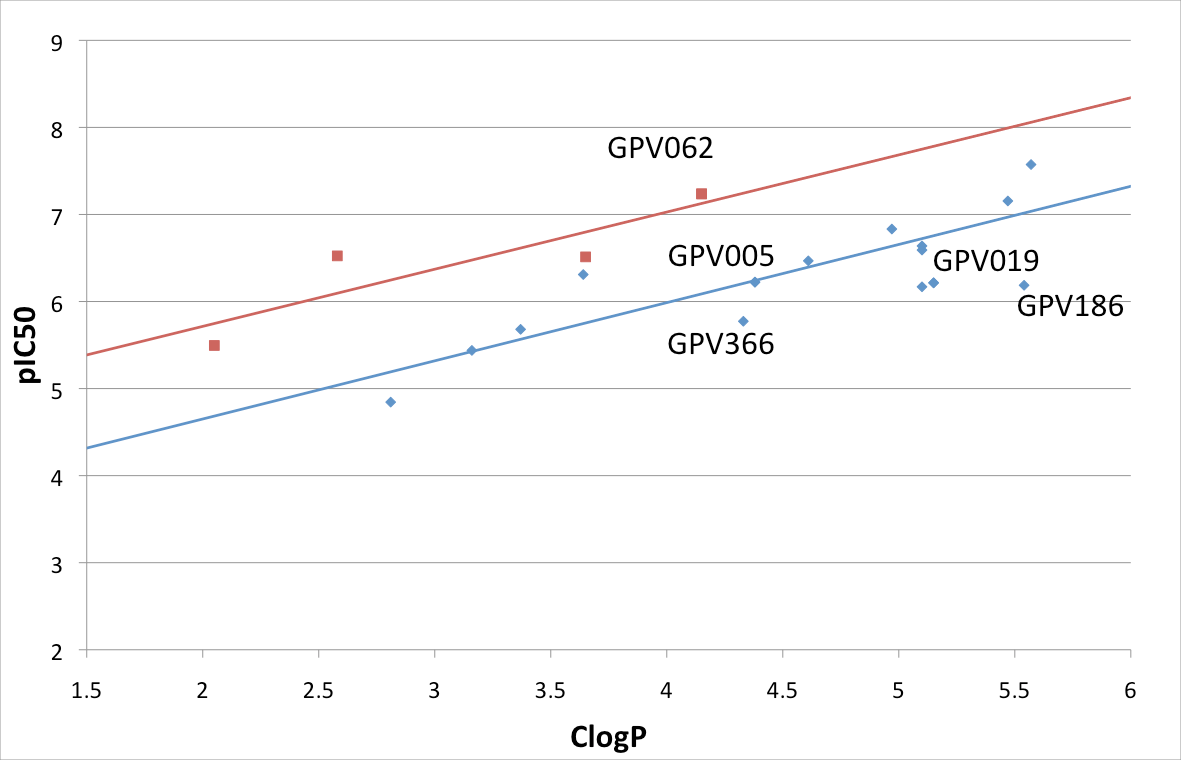

Supplement: Figure S1 — ClogP-pIC50 correlation of propafenone analogs. The ligands used for docking are highlighted. [24] (TIF) [file pcbi.1002036.s001.tif]

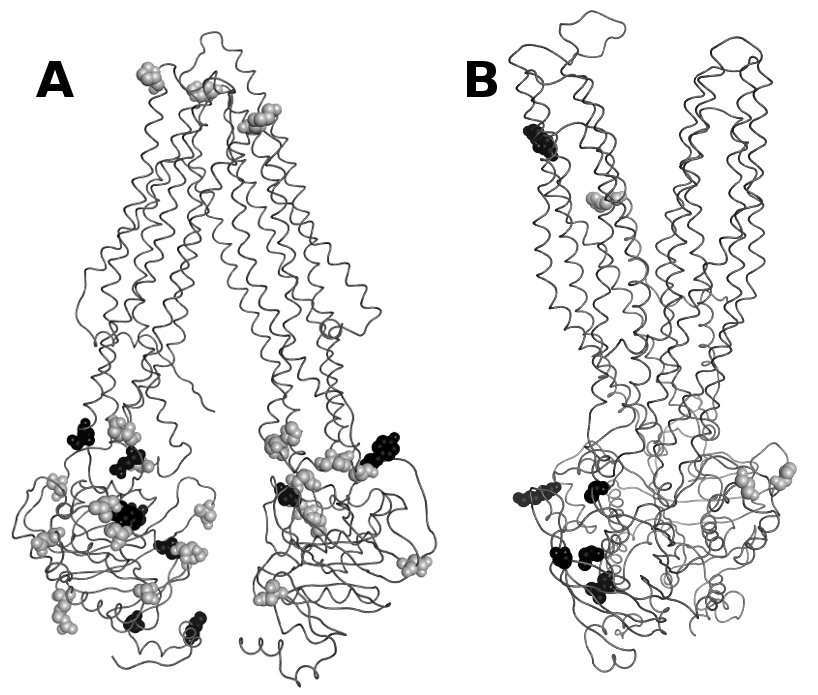

Supplement: Figure S2 — Outliers defined by PROCHECK analysis. A) 3G5U_Pgp, B) 2HYD_Pgp. Grey: generously allowed residues, black: disallowed residues. (TIFF) [file pcbi.1002036.s002.tiff]

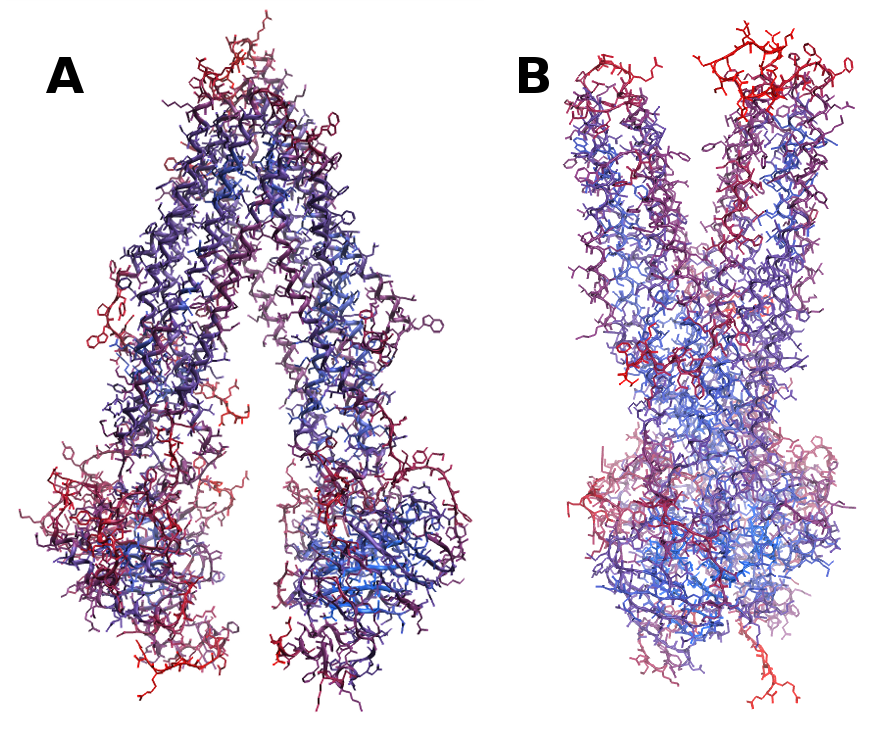

Supplement: Figure S3 — QMEAN analysis of the homology models generated with MODELLER. A) 3G5U_Pgp, B) 2HYD_Pgp. Blue: high quality regions, red: low quality regions. (TIFF) [file pcbi.1002036.s003.tiff]

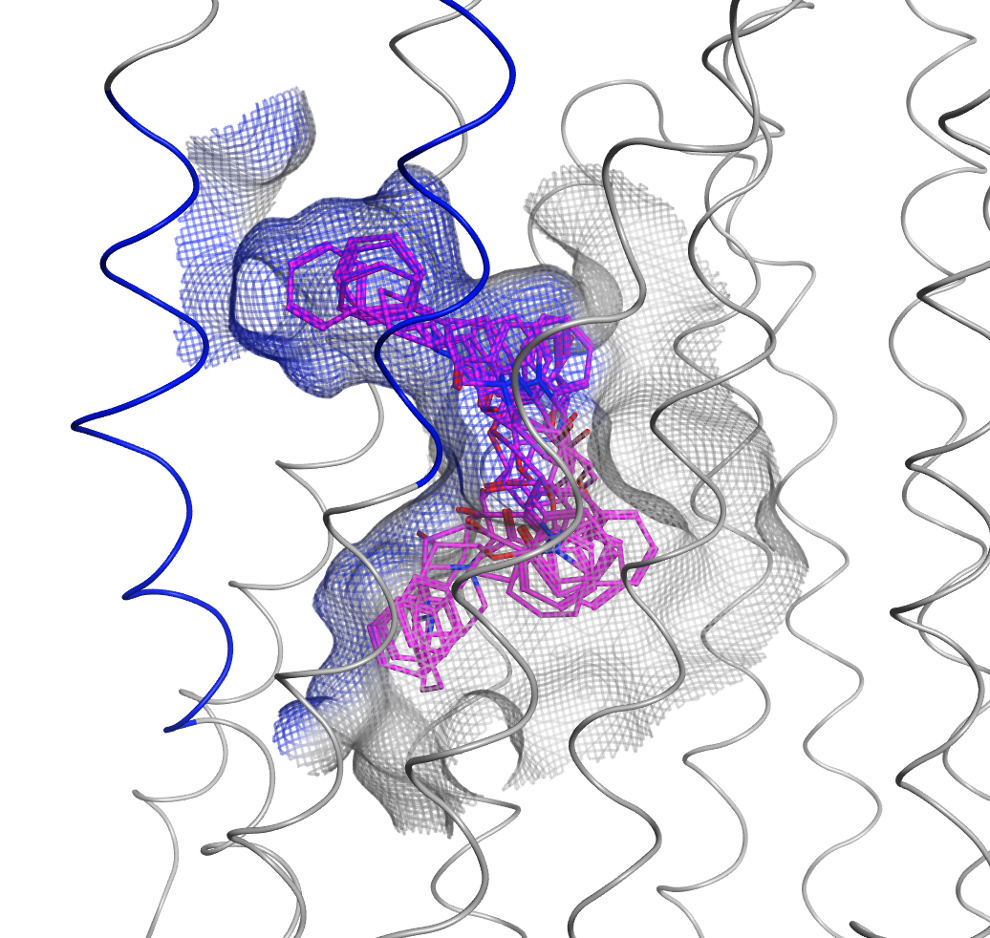

Supplement: Figure S4 — Common scaffold clusters after docking into 2HYD_Pgp. The blue surface indicates residues that are involved in propafenone binding, determined by photoaffinity labeling [14]. (TIFF) [file pcbi.1002036.s004.tiff]
