# Supplementary material for: Exhaustive Sampling of Docking Poses Reveals Binding Hypotheses for Propafenone Type Inhibitors of P-Glycoprotein
Source: PLoS Comput Biol. 2011 May 12;7(5):e1002036. doi: 10.1371/journal.pcbi.1002036 (PMC3093348; doi:10.1371/journal.pcbi.1002036)
Supplement: Figure S5 — Sequence alignment used for the generation of the homology model 3G5U_Pgp. The sequences of human P-gp and of the X-ray structure of mouse P-gp have been aligned as suggested by Aller et al. [12]. (PDF) [file pcbi.1002036.s005.pdf]

Alignment
Template: PDB code 3G5U (Chain A)
Target: human P-gp

Reference: Aller SG, Yu J, Ward A, Wenig Y, Chittaboina S, et al. (2009) Structure of P-glycoprotein reveals a molecular basis for poly-specific drug binding. Science 323: 1718-1722

```
3G5U_A.pdb      ---------------------------------VSVLTMFRYAGWLDRLYMLVGTLAAII 27
human_P-gp      MDLEGDRNGGAKKKNFFKLNNKSEKDKKEKKPTVSVFSMFRYSNWLDKLYMVVGTLAAII 60
                                                 ***::****:.***:***:********


3G5U_A.pdb      HGVALPLMMLIFGDMTDSFASVGNVS---KNSTNMSEADKRAMFAKLEEEMTTYAYYYTG 84
human_P-gp      HGAGLPLMMLVFGEMTDIFANAGNLEDLMSNITNRSDINDTGFFMNLEEDMTRYAYYYSG 120
                **..******:**:*** **..**:.   .* ** *: :. .:* :***:** *****:*


3G5U_A.pdb      IGAGVLIVAYIQVSFWCLAAGRQIHKIRQKFFHAIMNQEIGWFDVHDVGELNTRLTDDVS 144
human_P-gp      IGAGVLVAAYIQVSFWCLAAGRQIHKIRKQFFHAIMRQEIGWFDVHDVGELNTRLTDDVS 180
                ******:.********************::******.***********************


3G5U_A.pdb      KINEGIGDKIGMFFQAMATFFGGFIIGFTRGWKLTLVILAISPVLGLSAGIWAKILSSFT 204
human_P-gp      KINEGIGDKIGMFFQSMATFFTGFIVGFTRGWKLTLVILAISPVLGLSAAVWAKILSSFT 240
                ***************:***** ***:***********************.:*********


3G5U_A.pdb      DKELHAYAKAGAVAEEVLAAIRTVIAFGGQKKELERYNNNLEEAKRLGIKKAITANISMG 264
human_P-gp      DKELLAYAKAGAVAEEVLAAIRTVIAFGGQKKELERYNKNLEEAKRIGIKKAITANISIG 300
                **** *********************************:*******:***********:*


3G5U_A.pdb      AAFLLIYASYALAFWYGTSLVISKEYSIGQVLTVFFSVLIGAFSVGQASPNIEAFANARG 324
human_P-gp      AAFLLIYASYALAFWYGTTLVLSGEYSIGQVLTVFFSVLIGAFSVGQASPSIEAFANARG 360
                ******************:**:* **************************.*********


3G5U_A.pdb      AAYEVFKIIDNKPSIDSFSKSGHKPDNIQGNLEFKNIHFSYPSRKEVQILKGLNLKVKSG 384
human_P-gp      AAYEIFKIIDNKPSIDSYSKSGHKPDNIKGNLEFRNVHFSYPSRKEVKILKGLNLKVQSG 420
                ****:************:**********:*****:*:**********:*********:**


3G5U_A.pdb      QTVALVGNSGCGKSTTVQLMQRLYDPLDGMVSIDGQDIRTINVRYLREIIGVVSQEPVLF 444
human_P-gp      QTVALVGNSGCGKSTTVQLMQRLYDPTEGMVSVDGQDIRTINVRFLREIIGVVSQEPVLF 480
                ************************** :****:***********:***************


3G5U_A.pdb      ATTIAENIRYGREDVTMDEIEKAVKEANAYDFIMKLPHQFDTLVGERGAQLSGGQKQRIA 504
human_P-gp      ATTIAENIRYGRENVTMDEIEKAVKEANAYDFIMKLPHKFDTLVGERGAQLSGGQKQRIA 540
                *************:*************************:********************


3G5U_A.pdb      IARALVRNPKILLLDEATSALDTESEAVVQAALDKAREGRTTIVIAHRLSTVRNADVIAG 564
human_P-gp      IARALVRNPKILLLDEATSALDTESEAVVQVALDKARKGRTTIVIAHRLSTVRNADVIAG 600
                ******************************.******:**********************


3G5U_A.pdb      FDGGVIVEQGNHDELMREKGIYFKLVMTQT------------------------------ 594
human_P-gp      FDDGVIVEKGNHDELMKEKGIYFKLVTMQTAGNEVELENAADESKSEIDALEMSSNDSRS 660
                **.*****:*******:*********  **


3G5U_A.pdb      ---------------------------LDEDVPPASFWRILKLNSTEWPYFVVGIFCAII 627
human_P-gp      SLIRKRSTRRSVRGSQAQDRKLSTKEALDESIPPVSFWRIMKLNLTEWPYFVVGVFCAII 720
                                           ***.:**.*****:*** *********:*****


3G5U_A.pdb      NGGLQPAFSVIFSKVVGVFTNGGPPETQRQNSNLFSLLFLILGIISFITFFLQGFTFGKA 687
human_P-gp      NGGLQPAFAIIFSKIIGVFTRIDDPETKRQNSNLFSLLFLALGIISFITFFLQGFTFGKA 780
```

```
                          ********::****::****. . ***:************ *******************

3G5U_A.pdb                GEILTKRLRYMVFKSMLRQDVSWFDDPKNTTGALTTRLANDAAQVKGATGSRLAVIFQNI 747
human_P-gp                GEILTKRLRYMVFRSMLRQDVSWFDDPKNTTGALTTRLANDAAQVKGAIGSRLAVITQNI 840
                          *************:********************************** ******* ***

3G5U_A.pdb                ANLGTGIIISLIYGWQLTLLLLAIVPIIAIAGVVEMKMLSGQALKDKKELEGSGKIATEA 807
human_P-gp                ANLGTGIIISFIYGWQLTLLLLAIVPIIAIAGVVEMKMLSGQALKDKKELEGAGKIATEA 900
                          **********:*****************************************:*******

3G5U_A.pdb                IENFRTVVSLTREQKFETMYAQSLQIPYRNAMKKAHVFGITFSFTQAMMYFSYAACFRFG 867
human_P-gp                IENFRTVVSLTQEQKFEHMYAQSLQVPYRNSLRKAHIFGITFSFTQAMMYFSYAGCFRFG 960
                          ***********:***** *******:****:::***:*****************.*****

3G5U_A.pdb                AYLVTQQLMTFENVLLVFSAIVFGAMAVGQVSSFAPDYAKATVSASHIIRIIEKTPEIDS 927
human_P-gp                AYLVAHKLMSFEDVLLVFSAVVFGAMAVGQVSSFAPDYAKAKISAAHIIMIIEKTPLIDS 1020
                          ****:::**:**:*******:********************.:**:*** ****** ***

3G5U_A.pdb                YSTQGLKPNMLEGNVQFSGVVFNYPTRPSIPVLQGLSLEVKKGQTLALVGSSGCGKSTVV 987
human_P-gp                YSTEGLMPNTLEGNVTFGEVVFNYPTRPDIPVLQGLSLEVKKGQTLALVGSSGCGKSTVV 1080
                          ***:** ** ***** *. *********.*******************************

3G5U_A.pdb                QLLERFYDPMAGSVFLDGKEIKQLNVQWLRAQLGIVSQEPILFDCSIAENIAYGDNSRVV 1047
human_P-gp                QLLERFYDPLAGKVLLDGKEIKRLNVQWLRAHLGIVSQEPILFDCSIAENIAYGDNSRVV 1140
                          *********:**.*:*******:********:****************************

3G5U_A.pdb                SYEEIVRAAKEANIHQFIDSLPDKYNTRVGDKGTQLSGGQKQRIAIARALVRQPHILLLD 1107
human_P-gp                SQEEIVRAAKEANIHAFIESLPNKYSTKVGDKGTQLSGGQKQRIAIARALVRQPHILLLD 1200
                          * ************* **:***:**.*:********************************

3G5U_A.pdb                EATSALDTESEKVVQEALDKAREGRTCIVIAHRLSTIQNADLIVVIQNGKVKEHGTHQQL 1167
human_P-gp                EATSALDTESEKVVQEALDKAREGRTCIVIAHRLSTIQNADLIVVFQNGRVKEHGTHQQL 1260
                          *********************************************:***:**********

3G5U_A.pdb                LAQKGIYFSMVSVQA----- 1182
human_P-gp                LAQKGIYFSMVSVQAGTKRQ 1280
                          ***************
```
