# Supplementary material for: Exhaustive Sampling of Docking Poses Reveals Binding Hypotheses for Propafenone Type Inhibitors of P-Glycoprotein
Source: PLoS Comput Biol. 2011 May 12;7(5):e1002036. doi: 10.1371/journal.pcbi.1002036 (PMC3093348; doi:10.1371/journal.pcbi.1002036)
Supplement: Figure S6 — Sequence alignment used for the generation of the homology model of 2HYD_Pgp. The sequences of human P-gp and the bacterial ABC-exporter SAV1866 have been aligned as suggested by Stockner et al. [54]. (PDF) [file pcbi.1002036.s006.pdf]

Alignment
Template: PDB code 2HYD
Target: human P-gp

Reference: Stockner T, de Vries SJ, Bonvin AM, Ecker GF, Chiba P (2009) Data-driven homology modelling of P-glycoprotine in the ATP-bound state indicates fleibility of the transmembrane domains. FEBS J 276: 964-972

```
2HYD.pdb        --MIKRYLQFVKPYKYRIFATIIVGIIK-FGIPMLIPLLIKYAIDGVINNHA-------- 49
human_P-gp      VFSMFRYSNW--LDKLYMVVGTLAAIIHGAGLPLMMLVFGEMTDIFANAGNLEDLMSNIT 58
                   : ** ::    *  :..  :..**:  *:*::: :: : :   .  .:

2HYD.pdb        -------LTTDEKVHHLTIAIGIALFIFVIVRPPIEFIRQYLAQWTSNKILYDIRKKLYN 102
human_P-gp      NRSDINDTGFFMNLEEDMTRYAYYYSGIGAGVLVAAYIQVSFWCLAAGRQIHKIRKQFFH 118
                            ::..     .     :        :*:  :   ::.: ::.***::::

2HYD.pdb        HLQALSARFYA--NNQVGQVISRVINDVEQTKDFILTGLMNIWLDCITIIIALSIMFFLD 160
human_P-gp      AIMRQEIGWFD--VHDVGELNTRLTDDVSKINEGIGDKIGMFFQSMATFFTGFIVGFTRG 176
                 :   .  ::    ::**:: :*: :**.: :: *   :  :: .  *:: .: : *  .

2HYD.pdb        VKLTLAALFIFPFYILTVYVFFGRLRKLTRERSQALAEVQGFLHERVQGISVVKSFAIED 220
human_P-gp      WKLTLVILAISPVLGLSAAVWAKILSSFTDKELLAYAKAGAVAEEVLAAIRTVIAFGGQK 236
                 ****. * * *.  *:. *:   * .:* :.  * *:. .. .* : .* .* :*. :.

2HYD.pdb        NEAKNFDKKNTNFLTRALKHTRWNAYSFAAINTVTDIGPIIVIGVGAYLAISGSITVGTL 280
human_P-gp      KELERYNKNLEEAKRIGIKKAITANISIGAAFLLIYASYALAFWYGTTLVLSGEYSIGQV 296
                :* :.::*:  :    .:*::     *:.*   :   .  :.:  *: *.:**. ::* :

2HYD.pdb        AAFVGYLELLFGPLRRLVASFTTLTQSFASMDRVFQLIDEDYDIKNG-VGAQPIEIKQGR 339
human_P-gp      LTVFFSVLIGAFSVGQASPSIEAFANARGAAYEIFKIIDNKPSIDSYSKSGHKPDNIKGN 356
                 :..  : :   .: :  .*: ::::  .:  .:*::**:. .*..   ..:  :  :*.

2HYD.pdb        IDIDHVSFQYNDN-EAPILKDINLSIEKGETVAFVGMSGGGKSTLINLIPRFYDVTSGQI 398
human_P-gp      LEFRNVHFSYPSRKEVKILKGLNLKVQSGQTVALVGNSGCGKSTTVQLMQRLYDPTEGMV 416
                ::: :* *.* .. *. ***.:**.::.*:***:** ** **** ::*: *:** *.* :

2HYD.pdb        LIDGHNIKDFLTGSLRNQIGLVQQDNILFSDTVKENILLGRP--TATDEEVVEAAKMANA 456
human_P-gp      SVDGQDIRTINVRFLREIIGVVSQEPVLFATTIAENIRYGRE--NVTMDEIEKAVKEANA 474
                 :**::*: : .  **: **:*.*: :**: *: ***  **   ..* :*: :*.* ***

2HYD.pdb        HDFIMNLPQGYDTEVGERGVKLSGGQKQRLSIARIFLNNPPILILDEATSALDLESESII 516
human_P-gp      YDFIMKLPHKFDTLVGERGAQLSGGQKQRIAIARALVRNPKILLLDEATSALDTESEAVV 534
                :****:**: :** *****.:********::*** ::.** **:********* ***:::

2HYD.pdb        QEALDVLSKDRTTLIVAHRLSTITHADKIVVIENGHIVETGTHRELIAKQGAYEHLYSIQ 576
human_P-gp      QVALDKARKGRTTIVIAHRLSTVRNADVIAGFDDGVIVEKGNHDELMKEKGIYFKLVTMQ 594
                * ***   *.***:::******: :** *. :::* ***.*.* **: ::* * :* ::*

2HYD.pdb        NLMIKRYLQFVKPYKYRIFATIIVGIIK-FGIPMLIPLLIKYAIDGVINNHALTTDEKVH 635
human_P-gp      TA--WRIMKLNLTEWPYFVVGVFCAIINGGLQPAFAIIFSKIIGVFTRID----DPETKR 648
                .    * :::  .    :.. :: .**:    * :  :: *     .  :      *. :

2HYD.pdb        HLTIAIGIALFIFVIVRPPIEFIRQYLAQWTSNKILYDIRKKLYNHLQALSARFYA--NN 693
human_P-gp      QNSNLFSLLFLALGIISFITFFLQGFTFGKAGEILTKRLRYMVFRSMLRQDVSWFDDPKN 708
                : :  :.: :: : *:     *:: :    :.: :   :*  ::. :   .. ::   :*

2HYD.pdb        QVGQVISRVINDVEQTKDFILTGLMNIWLDCITIIIALSIMFFLDVKLTLAALFIFPFYI 753
human_P-gp      TTGALTTRLANDAAQVKGAIGSRLAVITQNIANLGTGIIISFIYGWQLTLLLLAIVPIIA 768
```

```
                 .* : :*: **. *.*. * : *  *  :  .:  .: * *: . :***  * *.*:

2HYD.pdb        LTVYVFFGRLRKLTRERSQALAEVQGFLHERVQGISVVKSFAIEDNEAKNFDKKNTNFLT 813
human_P-gp      IAGVVEMKMLSGQALKDKKELEGAGKIATEAIENFRTVVSLTQEQKFEHMYAQSLQVPYR 828
                ::  * :  *   : : .: *  .  :  * ::.: .* *:: *::  : : :.

2HYD.pdb        RALKHTRWNAYSFAAINTVTDIGPIIVIGVGAYLAISGSITVGTLAAFVGYLELLFGPLR 873
human_P-gp      NSLRKAHIFGITFSFTQAMMYFSYAGCFRFGAYLVAHKLMSFEDVLLVFSAVVFGAMAVG 888
                .:*::::  . :*:  :::  :.    : .****.    ::.  :  ... : :   .:

2HYD.pdb        RLVASFTTLTQSFASMDRVFQLIDEDYDIKNG-VGAQPIEIKQGRIDIDHVSFQYNDN-E 931
human_P-gp      QVSSFAPDYAKAKISAAHIIMIIEKTPLIDSYSTEGLMPNTLEGNVTFGEVVFNYPTRPD 948
                :: :  .  :::  *  ::: :*::   *..  . .   :  :*.: :..* *:*  . :

2HYD.pdb        APILKDINLSIEKGETVAFVGMSGGGKSTLINLIPRFYDVTSGQILIDGHNIKDFLTGSL 991
human_P-gp      IPVLQGLSLEVKKGQTLALVGSSGCGKSTVVQLLERFYDPLAGKVLLDGKEIKRLNVQWL 1008
                 *:*:.:.*.::**:*:*:** ** ****:::*: ****  :*::*:**::** : .  *

2HYD.pdb        RNQIGLVQQDNILFSDTVKENILLGRP--TATDEEVVEAAKMANAHDFIMNLPQGYDTEV 1049
human_P-gp      RAHLGIVSQEPILFDCSIAENIAYGDNSRVVSQEEIVRAAKEANIHAFIESLPNKYSTKV 1068
                * ::*:*.*: ***. :: ***  *    ..::**:*.*** ** * ** .**: *.*:*

2HYD.pdb        GERGVKLSGGQKQRLSIARIFLNNPPILILDEATSALDLESESIIQEALDVLSKDRTTLI 1109
human_P-gp      GDKGTQLSGGQKQRIAIARALVRQPHILLLDEATSALDTESEKVVQEALDKAREGRTCIV 1128
                *::*.:********::*** ::.:* **:********* ***.::*****   :.** ::

2HYD.pdb        VAHRLSTITHADKIVVIENGHIVETGTHRELIAKQGAYEHLYSIQNL---- 1156
human_P-gp      IAHRLSTIQNADLIVVFQNGRVKEHGTHQQLLAQKGIYFSMVSVQAGTKRQ 1179
                :******* :** ***::**:: * ***::*:*::* *  : *:*
```
